# Smoking, use of moist snuff and risk of celiac disease: a prospective study

**DOI:** 10.1186/1471-230X-14-120

**Published:** 2014-07-03

**Authors:** Jonas F Ludvigsson, Caroline Nordenvall, Bengt Järvholm

**Affiliations:** 1 Department of Medical Epidemiology and Biostatistics, Karolinska Institutet, Stockholm 171 77, Sweden; 2 Department of Paediatrics, Örebro University Hospital, Örebro University, Örebro, Sweden; 3 Department of Molecular Medicine and Surgery, Karolinska Institutet, Stockholm, Sweden; 4 Department of Public Health and Clinical Medicine, Umeå University, Umeå, Sweden

**Keywords:** Autoimmune, Coeliac, Gluten, Smoking, Moist snuff, Snus

## Abstract

**Background:**

Smoking status has been linked to several chronic inflammatory conditions but earlier research on smoking and celiac disease (CD) is contradictive. There are little data on moist snuff use and CD. The purpose of this study was to investigate the association between smoking, moist snuff use and later CD.

**Methods:**

We identified individuals with biopsy-verified CD (villous atrophy, histopathology stage Marsh III) through biopsy-reports from Sweden’s 28 pathology departments. Data on smoking and moist snuff were collected from the Swedish construction worker database “Bygghälsan” that includes preventive health care check-up data. Through poisson regression we calculated relative risks (RRs) for later CD according to smoking status (n = 305,722), and moist snuff status (n = 199,200) adjusting for age, sex and decade.

**Results:**

During follow-up 488 individuals with smoking data, and 310 with moist snuff data had a diagnosis of CD. The risk of CD was independent of smoking status with all RRs being statistically insignificant and ranging between 0.9 and 1.0. Compared to non-smokers, neither current smokers (RR = 0.93; 95% CI = 0.76-1.14) nor ex-smokers (RR = 0.98; 95% CI = 0.75-1.28) were at increased or decreased risk of CD. Risk estimates were similar in moderate smokers (RR = 0.92; 0.72-1.16) and heavy smokers (RR = 0.95; 0.74-1.24), and did not change when we examined the risk more than ten years after health examination (RR-moderate: 0.90; and RR-heavy: 0.95; both p > 0.05). Moist snuff use was not associated with later CD (RR = 1.00; 0.78-1.28), or with CD after more than ten years of follow-up (RR = 1.05; 0.80-1.38).

**Conclusions:**

We found no association between smoking, moist snuff use and future CD.

## Background

Celiac disease (CD) is characterized by small intestinal inflammation and is triggered by gluten exposure in genetically sensitive individuals [1]. CD occurs in 1-2% of the Western population [2,3], and has been linked to a number of disorders including type 1 diabetes [4], sepsis [5], lymphoproliferative malignancy [6], and excess mortality [7].

Although almost all individuals with CD are DQ2+ or DQ8+, genetic factors alone cannot explain the risk of CD [8]. Several environmental factors have therefore been put forward as explanations for CD (short breastfeeding [9], and to a lesser extent early infections [10,11] and elective caesarean section [12]), and stressful life events (such as pregnancy, death of relatives or surgery etc [13,14].

Most environmental risk factors have however only been studied in patients with childhood CD, and there is a lack of knowledge about CD risk factors in adults.

**Figure 1:**
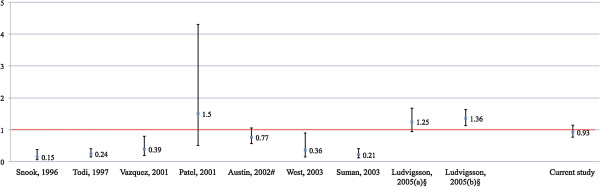
**Relationship between smoking and celiac disease (CD).** Relationship between smoking and celiac disease (CD). CD, celiac disease. Y-axis shows adjusted odds ratio/relative risk and 95% confidence intervals for CD in current smokers. Number of included patients with CD: Snook (n = 86) [22], Todi (n = 330) [21], Vazquez (n = 87) [19]; Patel (n = 82) [25], Austin (n = 430) [20], West (n = 87) [24], Suman (n = 138) [23], Ludvigsson (undiagnosed (a): n = 237; and diagnosed (b): n = 636) [26], and current study (n = 488). # The studies by Austin et al [20] and Thomasson et al [27] (not included in Figure 1) seem to be based on the same datasets. Thomasson et al do not present any odds ratio for CD among smokers but re-calculating their data we found a crude OR of 0.51, which is almost identical to that of Austin et al (crude OR = 0.52). For this reason the Thomasson et al study has not been included in Figure 1. § (a) association between smoking and undiagnosed CD; (b) association between smoking and diagnosed CD.

Smoking has been linked to a number of inflammatory disorders [15] including Crohn’s disease [16], and for a number of gastrointestinal disorders the association seems to be protective (e.g. ulcerative colitis [17] and sclerosing cholangitis [18]). Most earlier studies have shown an inverse relationship also between CD and smoking [19-24], but there are exceptions [25,26]. The largest study to date reported a positive association between smoking and CD [26] (Figure 1).

Smokeless tobacco (moist snuff) contains nicotine, which may influence the degree of intestinal inflammation. Moist snuff use in pregnant women has recently been linked to stillbirth risk [28], and older data suggest a positive association with inflammatory bowel disease [29]. We are unaware of any studies examining its association with CD.

In the current prospective study, we investigated the risk of CD among smokers and moist snuff users. Smoking and moist snuff are also contrasted as a means to disentangle the potential effect of nicotine exposure (occurs in both) on CD as opposed to effects of substances that only occur in tobacco smoke.

## Methods

Through the personal identity number [30] we linked data on smoking and moist snuff use obtained from some Swedish construction workers [31,32], with data on CD from Sweden’s 28 pathology departments [33].

### Smoking and moist snuff use

Between 1971 and 1993, results from health examinations of white- and blue-collar workers in the Swedish construction industry were computerized. The workers were offered preventive health check-ups by a national occupational health service, “Bygghälsan”. Data on health and exposures were collected through questionnaire, and a face-to-face interview by dedicated nurses. During 1975-77 no tobacco data were collected.

We categorized smoking into three groups (never, former, or current), and then subdivided current into moderate (≤14 cigarettes per day or equivalent), and heavy smokers (≥15 cigarettes per day). Moist snuff was divided into “ever” and “never users” for those with information after 1978. For the period 1971-74 only current snuff users were included as the variable for non-current users also included those who had not answered. Therefore, the analysis of moist snuff users was based on 199,185 persons (82,572 ever and 116,613 never users). Less than 10% of the ever-users reported that they had stopped using moist snuff. The quality of smoking data has been reviewed by Engholm et al and is high [34]. A previous review of smoking data at the first and second health examination (2–3 years apart) among 18,593 subjects in the “Bygghälsan” cohort found a 89% perfect match with data [35]. Inconsistencies regarding never-smoking status (study participants first indicated that they were current/former smokers in one questionnaire and then reported never-smoking in the second questionnaire) were reported for 2.7% [35].

The original cohort consisted of 389,132 individuals, 369,174 men and 19,418 women. Of those, 15 individuals were excluded because they had a diagnosis of CD before health examination. The mean year of birth in the remaining cohort was 1944 (range 1891-1976), mean year of first examination 1978.8 and mean age at first examination 34.6 years (range 14-82 years).

The number of recorded health examinations ranged from 1 to 15, with an average of 3. In this study we consistently used information on smoking and moist snuff from the first recorded visit (also defined as start of follow-up). There was information on tobacco smoking for 305,722 persons (290,449 men and 15,273 women). Information on moist snuff was available for 199,185 persons (187,338 men and 11,847 women).

Details of ethics approval: The study was approved by the Regional ethical review board in Umeå, 2013/225-31.

### Outcome measure: celiac disease

Between October 2006 and February 2008, we contacted all 28 Swedish pathology departments and obtained biopsy report data on duodenal and jejunal biopsies carried out in 1969-2008. IT personnel retrieved local data on date of biopsy, topography (duodenum or jejunum), morphology codes consistent with villous atrophy (histopathology stage Marsh III; see earlier paper for details [33]), as well as personal identity number [30]. Biopsy reports were on average based on 3 tissue specimen [36]. In this paper villous atrophy equals CD.

After removal of data irregularities, we had information on 29,148 individuals with CD [33]. After excluding two individuals with potentially incorrect date of birth, there remained 29,146 individuals. CD was the outcome measure of our study.

### Statistics

Person-years of follow up were calculated for each person from year of health examination within the construction worker service through 31 December 2008, CD, death or emigration, whichever occurred first. The person-years were stratified for age (10-year age-classes), decennium, sex and tobacco smoking or use of moist snuff.

In a post-hoc analysis we stratified for sex of the participants.

Relative risks were estimated from Poisson regression analysis. 95% confidence intervals were calculated by Wald estimates.

We used statistics software SAS® 9.3 to calculate statistics. P-values <0.05 were considered statistically significant.

A post-hoc power calculation (assuming a baseline prevalence of 0.16% for diagnosed CD among non-smokers) showed that we had an 80% power at 5% significance level to detect either a 29% increased risk or a 23% decreased risk of CD among smokers.

### Ethics

This study was approved by the Regional Ethical Review Board in Umeå (2013/225-31).

## Results

In the cohort of construction workers we identified 656 individuals (0.2%) with a biopsy-verified diagnosis of CD, 597 men and 59 women. They were on average 54.8 years old at time of diagnosis and the time between diagnosis and first health examination at “Bygghälsan” was 19.6 years (range 0-35 years). Of these 488 had available data on smoking status and 310 had data on moist snuff.

### Smoking and CD

The cases with CD in the cohort had similar distribution of smoking habits as the total cohort, (Table 1). The risk of CD was independent of smoking status (and time since health check-up) with all RRs being statistically insignificant and ranging between 0.9 and 1.0 (Table 2). Adjusted risk estimates were similar in moderate smokers (RR = 0.92; 95% CI = 0.72-1.16) and heavy smokers (RR = 0.95; 0.74-1.24), and did not change when we examined the risk more than ten years after health examination (RR-moderate: 0.90; and RR-heavy: 0.95; both p > 0.05) (Table 2). As some other studies have estimates for current smokers we have also calculated risk estimates for the combined group of moderate and heavy smokers (0 years after examination RR = 0.93; 95% CI 0.76-1.14 and 10-years after examination RR = 0.92; 95% CI. 0.74-1.15).

**Table 1:** Smoking habits in the cohort (N = 305,722 and cases with CD (N = 488)

**Smoking habits**	**Total cohort**	**CD cases**
	**(%)**	**(%)**
Non-smokers	43.6	44.7
Ex-smokers	15.1	16.1
Moderate smokers	24.2	22.5
Heavy smokers	17.1	16.7

**Table 2:** Relative risk of CD according to smoking status

**Smoking status**		
	**RR**^**#**^	**95% CI**^**§**^
Latency > =0 years*		
Non-smokers	1.00	
Ex-smokers	0.98	0.75-1.28
Moderate smokers	0.92	0.72-1.16
Heavy smokers	0.95	0.74-1.24
Latency > =10 years*		
Non-smokers	1.00	
Ex-smokers	0.99	0.75-1.31
Moderate smokers	0.90	0.70-1.16
Heavy smokers	0.95	0.72-1.27

RR, Relative risk adjusted for age, sex and decade.

1.00 constitutes reference.

*≥ years since health examination where the smoking habits were decided.

^#^Poisson regression analysis (age in 10-year-intervals).

^§^Wald estimates.

### Moist snuff use and CD

Moist snuff use was not associated with CD (RR = 1.00; 0.78-1.28). When restricting our analysis to follow-up after more than ten years after the health examination, the adjusted RR was 1.05 (95% CI = 0.80-1.38).

### Tobacco smoking and use of moist snuff and CD

We also compared the risk of CD in those who both smoked and used moist snuff compared with no use of tobacco products. The analysis was restricted to men with data on both smoking habits and use of moist snuff (N = 230,151) and showed no significant association between use of tobacco and never using moist snuff or smoking (RR = 0.91; 0.69-1.19).

### Posthoc analyses stratified for sex

A posthoc analysis restricted to only women showed an inverse risk with smoking (RR for current smokers 0.46 (0.22-0.96). An analysis restricted to only men showed no association with smoking (current smokers RR 0.98 95% CI 0.79-1.21).

## Discussion

This prospective cohort study found no association between smoking, moist snuff use and CD. In fact almost all RRs were between 0.9 and 1.0. Relative risks for future CD did not change when we required a 10-year-latency from health examination (with smoking status) and CD, and were similar in moderate (RR = 0.92) and heavy smokers (RR = 0.95). Also the RRs for CD in moist snuff users were around 1.

As can be seen from Figure 1, most earlier studies have shown an inverse relationship between CD and current smoking (none of them have looked at CD and moist snuff use). The exceptions are our previous study [26] and that of Patel et al. [25]. The current study found no association between CD and smoking, and shares several traits with our study from 2005 [26]. In both studies were data on smoking collected prospectively and independently of the CD diagnosis. The two studies are also the biggest so far (see legend, Figure 1) resulting in high study power and narrow confidence intervals. The 95% CI for CD in current smokers ranged between 0.76 and 1.14 in our present study.

As for ex-smoking and CD, all earlier studies have shown higher ORs than for current smoking (OR = 0.52 [23], 0.71 [24], 0.99 [20]; and in an Argentinean study the negative relationship with current smoking (OR = 0.39) even turned into a positive one for ex-smoking (OR = 1.46) [19]). The OR for ex-smoking and CD was almost identical to that of current smoking in the study from the Mayo clinic by Patel et al (1.6 [25]), and similarly in the current study where the ex-smoking RR was absolutely neutral (0.98).

As the collection of data on smoking and moist snuff was prospective and independent of that of CD, we are likely to have eliminated recall bias and selection bias with regards to controls. In contrast, several older studies have drawn controls from inpatients (ear-nose and ortopedics [23]; trauma and orthopedics [22]). This may well overestimate the smoking rate in controls since smokers are at increased risk of both ear-nose disease and fractures; and such controls subsequently underestimate the OR for CD among smokers. There is also a risk that some earlier studies have identified individuals with a more severe CD than in the average patient with CD since studies were limited to one (sometimes tertiary) centre [19,22,23,25], and this may bias the RRs in either direction. If the diagnosis of CD is depending on socioeconomic factors an inverse relation with smoking may occur as persons with low socioeconomic status usually smoke more today and may have lower availability to medical care in some countries. Earlier reports of CD and smoking have however shown that adjustment for socioeconomic status [22,23] rarely influences the risk estimates, and we recently showed that socioeconomic status and education level are only of minor importance for CD rates in Sweden [37]. The lack of association between CD and socioeconomic status in Sweden contrasts with British data, and given that some of the strongest inverse relationships between CD and smoking have been seen in British studies [20,22-24] we cannot rule out that country-specific factors and unidentified confounding have influenced risk estimates in our and earlier studies. This said socioeconomic status is unlikely to influence the current study more than marginally since our study includes workers with similar socioeconomic status for most occupational groups. However, we cannot fully rule out a healthy worker effect. If smoking is primarily associated with severe symptoms (classical CD [38]), and such symptoms prohibit individuals from working we may have underestimated an association (positive or negative) between CD and smoking.

In the current study, data on smoking were obtained through standardized questionnaires and sometimes with the assistance of nurses. Also earlier studies have used either questionnaires [20,24,25] or personal interviews [19,23] (or a combination [26,39]) to collect data.

The proportion of current smokers (41%) in our study was higher than in most earlier studies (proportion among controls: 10% [25], 13% [24], 24% (overall smoking rate [26]), 30% [23], 33% [19,20,22], and 42% [21]) but our rate is nevertheless consistent with Swedish national data from the 1970s-1990s. Of note, a high proportion of smokers will per se not influence RRs, but rather increase study power.

Some 95% of our study population consisted of males. This contrasts with other studies where women have dominated (females with CD: 56% [25], 66% [22,24], 67% [23], 73% [20], 83% [19], 100% [26]). Still, our study included more than 15,000 females. Females had higher risk of CD than men. The estimates in our study were otherwise adjusted for sex by a categorical variable. A posthoc analysis restricted to only women showed an inverse risk with smoking (RR for current smokers 0.46, while no inverse relationship was seen in men (current smokers RR 0.98). Women in this cohort include a rather large group of office workers. Analysing women adjusting for being an office worker or not showed a slightly lower RR (0.36; 95% CI 0.14-0.90) and separate analysis for the two groups of women showed similar results (RR = 0.36 and RR = 0.36) but with wider confidence limits. Thus, our data indicate that CD may be inversely associated to smoking in women but not in men. These findings contrast with our earlier study where we saw no relationship between smoking and CD in a female population [40]. One explanation for the difference between the two studies is that the former [40] was limited to women becoming pregnant (potentially different from the general CD population), but also that the CD diagnosis in our 2005 paper [40] was based on the inpatient diagnosis of CD, rather than biopsy-verified CD (current paper). Interaction between smoking and sex has been seen for other immune-mediated diseases such as rheumatoid arthritis [41]. Sex may also influence disease phenotype [42]. If smoking influences age at symptom onset that could also have influenced our findings since the median age at CD diagnosis was almost 55 years in this study, and our pregnancy-smoking paper was based on women aged 15-44 years [40], with most women being between 20 and 34 years old.

Finally, our cohort includes rather few women with CD (n = 43) and mostly non-smokers (n = 27) and fewer current smokers (n = 10) and ex-smokers (n = 6) so the negative relationship between CD and smoking may also be a chance finding. It should be stressed that the 95% CIs overlapped between men and women.

We did not have data on symptoms. We cannot rule out that if an inverse relationship with smoking is restricted to patients with classical [38] symptoms we might have missed it. For instance Snook et al [22] only included patients with diarrhoea and malabsorption. Considering that symptoms in adult CD seems to have changed [43,44], the relevance of studies restricted to patients with malabsorption can be questioned. When we reviewed patient charts from a random subset of individuals CD [33], diarrhoea occurred in 36% of our celiac patients, this is almost identical to that of patients with CD diagnosed later than year 2000 in a US centre (37%) [43]. Still we cannot rule out that the composition of celiac cases in our study differs slightly from that of studies where data collection on CD took place in the 1990s [19,20,23-25] or earlier [22], and that decade may have influenced our risk estimates. In our study, RRs generally increased (became “less inverse”) after adjustment for decade (data not shown).

In 2001, Prasad reported that anti-endomysial status may be important to smoking among patients with CD [45]. In their paper, EMA-negative status was strongly associated with smoking (OR = 7.0) [45]. The use of celiac serology to identify individuals with CD has increased greatly in the last 15-20 years, and it is possible that the association between CD and smoking is less inverse in serology-positive individuals. Results by West et al [24] argue against this as their celiac study population consisted of EMA-positive individuals. Although the current study did not require a positive celiac serology for the diagnosis of CD, some 88% with available data on serology were positive at time of small intestinal biopsy [33].

To our knowledge, this is the first prospective study on smoking, moist snuff use and CD. The RRs for CD among moist snuff users were consistently around 1, indicating that the nicotine levels of moist snuff are unlikely to protect against future CD.

This paper has some strengths and limitations. We identified individuals with CD using biopsy report data from 28 Swedish pathology departments and should hence have identified an average celiac population since more than 96% of all Swedish gastroenterologists and paediatricians biopsied their patients before CD diagnosis at the time of data collection [33].

When we validated 114 randomly selected patients using patient charts, we found that 95% of patients with villous atrophy had CD [33]. We also reviewed >1500 biopsy reports with villous atrophy and inflammation [33], and other diagnoses than CD were then very uncommon (the most common non-CD diagnosis recorded in biopsy reports with villous atrophy was IBD, seen in 0.3% of the reports) [33]. In other settings, other disorders such as autoimmune enteropathy, Whipple disease, common variable immunodeficiency, giardiasis and cow-milk protein intolerance should be considered in patients with villous atrophy [46].

A limitation of our study is that we did not screen for CD in our population. Considering that this study involved more than 300,000 individuals this was not feasible. Lack of serological screening might have been a problem if smoking was linked to CD with minor symptoms (and remaining undiagnosed) but considering that some of the lowest risk estimates for CD (OR = 0.15) was seen in the study by Snook et al [22] where all patients suffered from malabsorption and diarrhoea this is unlikely to be the case. In a subset of patients where we had access to patient chart data [33], some 79% of CD patients had gastrointestinal symptoms.

An additional study limitation is that we were unable to identify individuals who might have quit smoking after the first data collection. Finally, despite the large number of study participants we cannot rule out a minor inverse or positive association between CD and smoking due to limited study power.

Smoking influences the immune system and more specifically may decrease the intestinal permeability [47]. This could potentially protect against gliadin exposure and risk of CD, and has been argued as the cause for earlier findings of an inverse relationship between smoking and CD. If smoking protects against small intestinal inflammation our data argue that substances that only occur in tobacco smoke are unlikely to explain an inverse relationship with CD since risk estimates in our study were almost identical in smokers and moist snuff users. However, the most likely explanation for RRs around 1 in both smokers and moist snuff users are that these factors do not play a major role in the aetiology of CD in a Swedish setting.

## Conclusions

In conclusion, we found no association between smoking, moist snuff use and CD.

## Abbreviations

CD: Celiac disease; CI: Confidence interval; RR: Relative risk.

## Competing interests

The authors (JFL, CN, and BJ) declare that they have no conflicts of interest.

## Authors’ contributions

ICMJE criteria for authorship read and met: JFL, CN and BJ. Agree with the manuscript’s results and conclusions: JFL, CN and BJ. Designed the experiments/the study: JFL and BJ. Collected data: JFL and BJ. Analyzed the data: BJ. Wrote the first draft of the paper: JFL. Contributed to the writing of the paper: CN and BJ. Contributed to design of study and interpretation of the data analyses: JFL, CN and BJ. Interpretation of data and approved the final version of the manuscript: JFL, CN and BJ. Responsible for data integrity: JFL and BJ. Supervised the project: JFL and BJ. Obtained funding: JFL and BJ. All authors read and approved the final manuscript.

## Pre-publication history

The pre-publication history for this paper can be accessed here:

http://www.biomedcentral.com/1471-230X/14/120/prepub
